# Supplementary material for: Delusion progression process from the perspective of patients with psychoses: A descriptive study based on the primary delusion concept of Karl Jaspers
Source: PLoS One. 2021 Apr 27;16(4):e0250766. doi: 10.1371/journal.pone.0250766 (PMC8078756; doi:10.1371/journal.pone.0250766)
Supplement: S1 Text — (DOCX) [file pone.0250766.s001.docx]

# Delusion (Special thought) and its Origin Assessment Interview (DOAI)

“Now, I am going to ask you about some special thoughts you may have had in the last three months, which you are concerned about, distressed with, etc., and to request to think about the origins of the thoughts: how the thoughts were formed or what made you have the thoughts.”

“The doctor in charge of you has informed us that you had the following thoughts. Will you please explain these in brief?”

1.

2.

3.

“We refer to the thoughts as [(Special thoughts)] (e.g., ‘Government conspiracy,’ ‘Unreasonable attacks,’ ‘God's blessings,’ ‘Sinfulness’) in this interview from now on.”

## A. Themes of the thoughts

A-1. (Persecutory delusions)

“Have you thought that some person or organization is trying to hurt you, following you, or working against you?”

“Are there persons or an organization that has unreasonable hostility or bad will against you?”

Statements about experiences of being hurt, bad will, etc.:

Persecutory delusions

| 1. Absent or minimal: Within the upper extreme of normal limits | 2. Mild: Unrealistic concerns about being persecuted or poorly formed delusions, without distinctive influence on behavior or social interactions | 3. Moderate: Some persecutory delusions, with some influence on behavior and social interactions | 4. Moderately severe: Several apparent persecutory delusions, with substantial influence on behavior and social interactions | 5. Severe: Many firmly formed persecutory delusions, domineering behavior and social interactions |
| --- | --- | --- | --- | --- |

A-2. (Grandiose delusions)

“Have you thought that you are especially important in some way, or have special powers, knowledge, or importance?”

Statements of special importance or powers:

Grandiose delusions

| 1. Absent or minimal: Within the upper extreme of normal limits | 2. Mild: Unrealistic expansiveness or boastfulness or poorly formed delusions, without distinctive influence on behavior or social interactions | 3. Moderate: Some grandiose delusions with some influence on behavior and social interactions | 4. Moderately severe: Several apparent grandiose delusions with substantial influence on behavior and social interactions | 5. Severe: Many firmly formed grandiose delusions, domineering behavior and social interactions |
| --- | --- | --- | --- | --- |

A-3. (Hypochondriacal delusions)

“Have you thought that you are suffering from very grave or fatal physical illness? What diagnosis and treatment have you received in a hospital?”

Statements about illness and treatment:

Hypochondriacal delusions

| 1. Absent or minimal: Within the upper extreme of normal limits | 2. Mild: Unrealistic concerns about health and somatic issues or poorly formed delusions, without distinctive influence on behavior or social interactions | 3. Moderate: Some hypochondriacal delusions, with some influence on behavior and social interactions | 4. Moderately severe: Several apparent hypochondriacal delusions, with substantial influence on behavior and social interactions | 5. Severe: Many firmly formed hypochondriacal delusions, domineering behavior and social interactions |
| --- | --- | --- | --- | --- |

A-4. (Delusions of assistance or being loved (erotomania))

“Are there people who are trying to help you in a special way?”

“Is there some person who is in love with you and express his or her affection in an unusual way?”

“Do you have a ‘secret lover’ who, when you have contacted them, denied that they are in love with you?”

Note: If it is maintained that Subject has, implicitly or explicitly, been blamed or threatened because of the love relationship, it is also to be evaluated as a persecutory delusion in Item A-1.

Statements about the relationship:

Delusions of being assisted or loved (erotomania)

| 1. Absent or minimal: Within the upper extreme of normal limits | 2. Mild: Some unrealistic ideas of being loved or assisted, or poorly formed delusions, without distinctive influence on behavior or social interactions | 3. Moderate: Some delusions of being assisted or loved, with some influence on behavior or social interactions | 4. Moderately severe: Several apparent delusions of being assisted or loved, with substantial influence on behavior and social interactions | 5. Severe: Many firmly formed delusions of being assisted or loved, domineering, behavior and social interactions |
| --- | --- | --- | --- | --- |

A-5. (Delusions of guilt)

“Have you thought that you have committed a crime or sin, or have been accused of it?”

“Do you need to be punished?”

“Do you feel worthless, sinful, or guilty?”

Statements about the crime or sin:

Delusions of guilt

| 1. Absent or minimal: Within the upper extreme of normal limits | 2. Mild: Unrealistic ideas of guilt or self-depreciation, or poorly formed delusions without distinctive influence on behavior or social interactions | 3. Moderate: Some delusions of guilt, with some influence on behavior or social interactions | 4. Moderately severe: Several apparent delusions of guilt, with substantial influence on behavior and social interactions | 5. Severe: Many firmly formed delusions of guilt, domineering behavior and social interactions |
| --- | --- | --- | --- | --- |

A-6. Other themes

Check all that apply.

1. □ Delusions of poisoning

2. □ Delusions of immortality

3. □ Descent delusions

4. □ Delusions of non-existence

5. □ Delusions of jealousy

6. □ Delusions of death of relatives

7. □ Delusions of theft (things being stolen)

8. □ Delusions of pregnancy

9. □ Delusions of poverty

10. □ Parasite delusions

Statements about these themes:

## B. Origins of the thoughts (Reasons for developing the thoughts)

(Primary delusional experiences (PDEs))

“Are there some origins of [(Special thoughts)]?”

“Can you explain how and why the [(Special thoughts)] developed?”

“Are there particular events or experiences that caused you to get [(Special thoughts)]?”

1.

2.

3.

B-1. (Delusional perception)

“Are the origins of [(Special thoughts)] particular things or events you experienced or what you saw, heard, or sensed in a certain situation?”

“Have you heard or seen someone talk about you or someone sending a special message to you through gestures or other signs?”

“How often do you have that kind of experience?”

Note: The experiences addressed in this item (B-1) involve a normal percept, which is produced on the basis of actual perceptual experience, that has a special meaning for Subject. E.g., “I saw two passers-by who laughed while talking to each other. Though I could not hear well what they were talking about, I knew that they had mocked me.”

Delusions of reference are also evaluated here if they appear to be initiated by or closely connected with particular perceptual experiences.

Statements about delusional perception:

Level of explanation by delusional perception as an origin (B-1)

| 1. None | 2. Unclear: Explained only vaguely as an origin | 3. Mild:  Explained as a possible origin | 4. Moderate:  Explained as one of some origins or a probable origin | 5. High:  Explained definitely as the major origin |
| --- | --- | --- | --- | --- |

Frequency of delusional perception (B-1-2)

| 1.None or very rare | 2. Rare: Approx. once in a day | 3. Sometimes: 。  Some (3-6) times in a day | 4.Often: Approx. 10 times a day | 5. Almost always |
| --- | --- | --- | --- | --- |

B-2 (Delusional memories)

“Are the origins of [(Special thoughts)] memories of past events (ones prior to the past three months)?”

“How often do you recall that memory?”

Nate: Both memories of usual (ordinary) contents and of unusual (abnormal) contents are to be rated in this item, B-2. In addition, they also need to be differentiated from the perceptual experiences within the past three months, which are to be rated in Item B-1 or B-2.

Statements about the memories:

Level of explanation by delusional memories as origins (B-2)

| 1. None | 2. Unclear: Explained only vaguely as an origin | 3. Mild:  Explained as a possible origin | 4. Moderate:  Explained as one of some origins or a probable origin | 5. High:  Explained definitely as the major origin |
| --- | --- | --- | --- | --- |

Frequency of delusional memory recall (B-2-2)

| 1.None or very rare | 2. Rare: Approx. once in a day | 3. Sometimes: 。  Some (3-6) times in a day | 4.Often: Approx. 10 times a day | 5. Almost always |
| --- | --- | --- | --- | --- |

B-3. (Delusional mood or atmosphere)

“Does the origin of [(Special thoughts)] involve experiencing a state of uneasy mood or atmosphere, in which you felt as though the circumstance is strange, threatening, or has ominously changed?”

“Have you experienced a sense of impending doom that something awful will happen, but you do not know what?”

“How often do you have that kind of experience?”

Note: Experiences referred to as “world destruction phantasy or delusion of catastrophe (Weltuntergangserlebnis)” are also assessed in this item when they are not clearly connected with any perceptional experience.

If some distinctive perceptual experiences are counted as origins by Subjects, they should also be scored in Item B-1.

Statements about delusional mood or atmosphere:

Level of explanation by delusional mood or atmosphere as origins. (B-3)

| 1. None | 2. Unclear: Explained only vaguely as an origin | 3. Mild:  Explained as a possible origin | 4. Moderate:  Explained as one of some origins or a probable origin | 5. High:  Explained definitely as the major origin |
| --- | --- | --- | --- | --- |

Frequency of delusional mood or atmosphere (B-3-2)

| 1.None or very rare | 2. Rare: Approx. once in a day | 3. Sometimes: 。  Some (3-6) times in a day | 4.Often: Approx. 10 times a day | 5. Almost always |
| --- | --- | --- | --- | --- |

B-4. (Delusional intuition)

“Have you got the thoughts in the form of a sudden notion, which gave a new color and meaning to your experience, like an intuition, revelation, or prognostication? Is it sure that the notion is not related to what you heard, saw, sensed, or recalled?”

“How often do you have that kind of experience?”

Note: Experiences described as “Autochthonous ideas” and “Wahneinfall” are also rated in Item B-4.

If there involved any percept, memory recall or mood (atmosphere) in the evolution of the thoughts, they should be considered to be evaluated in respective Items of B-1, B-2 and B-3. Likewise, if any experiences of hallucination or self-disturbances are involved, this item should not be rated positively.

Statements about delusional intuition:

Level of explanation by delusional intuition (B-4)

| 1. None | 2. Unclear: Explained only vaguely as an origin | 3. Mild:  Explained as a possible origin | 4. Moderate:  Explained as one of some origins or a probable origin | 5. High:  Explained definitely as the major origin |
| --- | --- | --- | --- | --- |

Frequency of delusional intuition (B-4-2)

| 1.None or very rare | 2. Rare: Approx. once in a day | 3. Sometimes: 。  Some (3-6) times in a day | 4.Often: Approx. 10 times a day | 5. Almost always |
| --- | --- | --- | --- | --- |

## C. Responses and judgements of Subjects

C-1. Affective responses

C-1-a. (Unpleasant feeling)

“Do [(Special thoughts)] arouse an unpleasant feeling in you? Or, Do [(Special thoughts)] make you comfortable?”

“If you feel unpleasant while thinking of [(Special thoughts)], what degree of unpleasantness do you have?”

Statements about unpleasant feelings (or comfort) aroused by thinking of the thoughts:

Level of unpleasantness

| 1. Opposite: Feeling comfortable | 2. Neutral: Neutral or unable to answer | 3. Mild: Feeling mildly unpleasant | 4. Moderate: Feeling moderately unpleasant | 5. Severe: Feeling greatly unpleasant |
| --- | --- | --- | --- | --- |

C-1-b. (Anxiety and tension)

“Do [(Special thoughts)] arouse anxiety and tension in you? Or, Do [(Special thoughts)] relax you?”

“If you feel anxious and tense while thinking of [(Special thoughts)], what degree of anxiety and tension do you have?”

Statements about anxiety and tension (or relaxation) aroused by thinking of the thoughts:

Level of anxiety and tension

| 1. Opposite: Feeling relaxed | 2. Neutral or unable to answer | 3. Mild: Feeling mildly anxious and tense | 4. Moderate: Feeling moderately anxious and tense | 5. Severe: Feeling greatly anxious and tense |
| --- | --- | --- | --- | --- |

C-1-c. (Excitement and anger)

“Do [(Special thoughts)] arouse excitement and anger? Or, Do [(Special thoughts)] calm you down?”

“If you feel excitement and anger while thinking of [(Special thoughts)], what degree of excitement and anger do you have?”

Statements about excitement and anger (or calm) aroused by thinking of the thoughts:

Level of excitement and anger

| 1. Opposite: Feeling calmed down | 2. Neutral: Neutral or unable to answer | 3. Mild: Feeling mildly excited and angered | 4. Moderate: Feeling moderately excited and angered | 5. Severe: Feeling greatly excited and angered |
| --- | --- | --- | --- | --- |

C-2. (Preoccupation)

“To what degree are you preoccupied with [(Special thoughts)] in your daily life?”

Statements about preoccupation:

Level of preoccupation

| 1. None: Not preoccupied | 2. Slight: A little preoccupied with slight influence on daily activities | 3. Mild: Mildly preoccupied with some influence on daily activities | 4. Moderate: Moderately preoccupied with substantial influence on daily activities | 5. Severe: Greatly preoccupied, affecting almost all daily activities |
| --- | --- | --- | --- | --- |

C-3 (Inability to stop thinking of the thoughts and distracting himself/herself)

“Can you freely stop thinking of [(Special thoughts)], and start thinking of other things?”

“Do you have difficulty in stopping thinking of [(Special thoughts)]?”

Statements about inability to stop thinking of the thoughts:

Level of inability to stop thinking

| 1. None: Able to stop thinking easily | 2. Slight: Able to stop thinking with a little effort | 3. Mild: A little difficult to stop thinking, but manage to stop | 4. Moderate: Fairly difficult to stop thinking, or sometimes requiring great effort to stop | 5. Severe: Greatly difficult to stop thinking, or often unable to stop |
| --- | --- | --- | --- | --- |

C-4. (Distress caused by the thoughts)

“How much distress or trouble do [(Special thoughts)] cause you?”

“To what degree are you distressed by thinking of [(Special thoughts)]?”

Statements about distress caused by thinking of the thoughts:

Level of distress

| 1. None: Not distressed | 2. Slight: A little distressed | 3. Mild: Mildly distressed | 4. Moderate: Moderately distressed | 5. Severe: Greatly distressed |
| --- | --- | --- | --- | --- |

C-5. (Pervasiveness in areas of daily activities)

“Are your usual daily activities such as reading books and watching TV disturbed by [(Special thoughts)]?”

“How pervasively is your daily life affected by [(Special thoughts)]?”

“Can you count the areas of your daily activities that are affected by [(Special thoughts)]?”

Statement about pervasiveness:

Level of pervasiveness

| 1. None or minimal: Not affecting daily activities or having utterly encapsulated delusions | 2. Slight: Affecting few areas of daily activities | 3. Mild: Affecting some areas of daily activities | 4. Moderate:  Affecting several areas of daily activities | 5. Severe:  Affecting many areas of daily activities |
| --- | --- | --- | --- | --- |

C-6. (Delusional system formation (delusional elaboration/explanation))

“Do you think that there are specific meanings or backdrops concerning [(Special thoughts)]?”

“Can you point out more reasons why the particular events or experiences are caused?”

“Can you further explain about them?”

Statements about systematization: Conspiracy – trial – persecution – politics

Level of delusional system formation

| 1. None:  No delusional elaboration, or within the limit of ordinary explanation | 2. Slight: Few delusional elaborations of experiences and events, mildly connected to the thoughts | 3. Mild: Some delusional elaborations of experiences and events, clearly connected to the thoughts | 4. Moderate: Several delusional elaborations, incorporated into a delusional system | 5. Severe: Many delusional elaborations, incorporated into a firmly formed delusional system |
| --- | --- | --- | --- | --- |

C-7. Judgment (recognition) about the thoughts

C-7-a. (Conviction of delusion)

“Are you really sure of [(Special thoughts)]? Have you ever cast a doubt on [(Special thoughts)]?”

Statements about conviction:

Level of conviction

| 1. None: No conviction | 2. Slight: Not sure, only suggesting | 3. Mild: Probably sure, but with some doubt | 4. Moderate: Fairly sure, but with slight doubt | 5. Severe: Absolutely sure |
| --- | --- | --- | --- | --- |

C-7-b. (Sharing of the thoughts with others)

“Are [(Special thoughts)] shared with other people?”

“To what degree do you think that other people can share [(Special thoughts)]?”

Statements about sharing of the thoughts with others:

Level of sharing of the thoughts with others

| 1. None: Not shared | 2. Slight: Possibly shared with a few people | 3. Mild: Probably shared with some people | 4. Moderate: Fairly shared with several people | 5. Severe: Surely shared with many people |
| --- | --- | --- | --- | --- |

C-7-c. (Attribution of the thoughts to reality)

“Do you think that [(Special thoughts)] are derived from the reality or your imagination?”

“What percentage of your imaginations or expectations is included in [(Special thoughts)]?”

Statements about attribution of the thoughts to reality (or involvement of imagination):

Level of reality attribution (denying imagination involvement)

| 1. None: Denying the reality of thoughts, or attributing to imagination | 2. Slight: Admitting probable involvement of imagination, doubting the reality of thoughts | 3. Mild : Principally attributing thoughts to the reality, but admitting involvement of imagination | 4. Moderate: Clearly attributing thoughts to the reality, but admitting the possibility of imagination involvement | 5. Severe: Totally attributing thoughts to the reality, or firmly denying the involvement of imagination |
| --- | --- | --- | --- | --- |

C-7-d. (Denying mental illness involvement)

“Do you think that [(Special thoughts)] are due to a certain mental illness or not?”

“What degree of relatedness with mental illness do [(Special thoughts)] have?”

Statements about mental illness involvement:

Level of denying mental illness involvement

| 1. None or minimal: Principally admitting mental illness involvement | 2. Mild: Admitting some involvement of mental illness, but denying its importance in the thoughts | 3. Moderate: Principally denying mental illness involvement, but admitting its possibility | 4. Moderately severe: Clearly denying attribution to mental illness, hardly admitting its involvement in the thoughts | 5. Severe: Totally denying attribution to mental illness, or firmly denying involvement of mental illness in the thoughts |
| --- | --- | --- | --- | --- |
