# Supplementary material for: Delusion progression process from the perspective of patients with psychoses: A descriptive study based on the primary delusion concept of Karl Jaspers
Source: PLoS One. 2021 Apr 27;16(4):e0250766. doi: 10.1371/journal.pone.0250766 (PMC8078756; doi:10.1371/journal.pone.0250766)
Supplement: Attachment — Submitted filename: Response letter to the decision.docx [file pone.0250766.s004.docx]

PONE-D-20-21497

Delusion progression process from the perspective of patients with psychoses: A descriptive study based on the primary delusion concept of Karl Jaspers (Formerly, Delusion progression process viewed from the primary delusion concept of Karl Jaspers)

Responses to requirements of the editor and comments of the reviewers, and changes made on that basis are listed below.

**Requests from the editor (Journal Requirements)**

**Requirement 1**

Please ensure that your manuscript meets PLOS ONE's style requirements, including those for file naming. The PLOS ONE style templates can be found at <https://journals.plos.org/plosone/s/file?id=wjVg/PLOSOne_formatting_sample_main_body.pdf> and <https://journals.plos.org/plosone/s/file?id=ba62/PLOSOne_formatting_sample_title_authors_affiliations.pdf>

**Response to Requirement 1**

We have amended the manuscript according to the PLOS ONE style requirements: having renamed supporting files, adding citation of a supporting file, and adding continuous line numbers.

**Requirement 2**

Please describe in your methods section how capacity to consent was determined for the participants in this study.

**Response to Requirement 2**

We have added a criterion that had been defined in the protocol of this study, and applied during the enrollment of candidate patients as follows (underlined part) (Line 155-157 in Method section).

“The criteria for candidate patients were (i) having a persisting delusion of a mild or more severe level rated in Item P1. Delusion of Positive and Negative Syndrome Scale (PANSS), [32] (ii) being clinically judged to be stable enough to undergo the assessment not having apparent difficulties in ordinary interpersonal communication and personal care, (iii) not having a prominent organic cognitive disorder or intellectual disability that made the assessment difficult, and (iv) providing consent to participate in this study. Investigators confirmed that subjects fulfilled these criteria and received written informed consent for participation. ”

The criterion added here had also been written in the request letter for physicians in charge to list candidate patients.

**Requirement 3**

Please modify the title to ensure that it is meeting PLOS’ guidelines (https://journals.plos.org/plosone/s/submission-guidelinesloc-title). In particular, the title should be "specific, descriptive, concise, and comprehensible to readers outside the field" and in this case it is not informative and specific about your study's scope and methodology.

**Response to Requirement 3**

We have replaced the former title with the new one, “Delusion progression process from the perspective of patients with psychoses: A descriptive study based on the primary delusion concept of Karl Jaspers” to indicate more clearly the methodological position and the scope of this study.

**Response to reviewers’ comments**

**Reviewer 1**

**General comment**

… However, I have real difficulties with the interpretative scope of the results described, and more precisely with the definition of primary delusions (PD) by Karl Jaspers. Before proposing a study to “well-verify” the Jaspersian hypothesis, it is advisable, in my opinion, to define it in more detail (introduction and discussion) and more correctly. If my understanding of Jaspers' specifically schizophrenic delusional processes is correct (especially in” Causal and Understandable Relationships” section), PD experiences involve several points:…

**Response to Reviewer 1’s general comment**

First of all, we would like to present our general revision policy for this manuscript since the reviewer 1’s comments are intrinsically related to each other. The policy is that the methodology and the scope of this study are to be clarified: addressing the experiences of patients with delusion by the method of descriptive psychopathology, and exploring delusion progression processes on the basis of patients’ self-reflections.

In this revision, we firstly have added descriptions of the axial points of Jaspersian methodology concerning PD concept in addition to the method of subjective experience description: the introduction of un-understandability (incomprehensibility) from the second (intersubjectve) and/or the third person perspective, and the special attention to abnormal forms of experiences such as self-evidentness of PD experiences. These methods have briefly been presented in the introduction section of this manuscript.

Subsequently, we have clarified the position of this study that basically dealt with phenomena of delusion from the first person perspective while other Jaspersian approaches counted above are not placed in the front. The position of this study is situated in “descriptive psychopathology” defined by Stanghellini, G (“The meanings of psychopathology” Current Opinion Psychiatry 2009). Therefore, the meaning of “verification” the reviewer questioned concerns solely the descriptive approach from the first person perspective.

The corrections made according to the revision policies are presented below, excepting those concerning un-understandability that are shown in Response to Reviewer 1’s comment A.

**Title:** The title has been changed to indicate more clearly the methodological position and the scope of this study.

“Delusion progression process from the perspective of patients with psychoses: A descriptive study based on the primary delusion concept of Karl Jaspers”

**Line 25–27 in Abstract:**

Although the primary delusion (PD) concept of Karl Japers (1913) is a classical hypothesis in descriptive phenomenology concerning primordial experience of delusion, to our knowledge, it has not been verified in empirical studies of patients with psychoses,

**Line 141–143 in Introduction section:**

Based on the method of describing patients’ subjective experiences and self-reflections, though it is only a restricted part of the methodology of Jaspers, the present study attempted to verify whether PDs were initial phenomena of delusional experiences.

**Line 484–485 in Discussion section:**

By examining the subjects’ self-reflections about origins of delusional experience, the present study demonstrated that the majority of subject patients recognized PDs as origins of delusions and were concurrently experiencing them.

**Line 596–598 in Conclusions subsection:**

This study elucidated the principal position of PDs as origins of delusions and their close relationship with the delusion characteristics in self-reflections of patients with psychoses, which has confirmed the hypothesis set up by Karl Jaspers over a hundred years ago.

Responses to Individual comments are presented below.

**Comment A**

A break in understanding (incomprehensibility), whether historical, or biographical (through the sequencing of experiences, by “genetic” understanding) or more immediate and empathetic (through “static” understanding),

**Response to Reviewer 1’s comment A**

We agree that the introduction of (un-)understandability is one of the axial approach in Jaspersian psychopathology. We have added the explanation of the position of this study concerning the un-understandability in the manuscript.

**Line 63–72 in Introduction section:**

Among all, Jaspers is the author who laid down the methodology of the phenomenological approach for in-depth description of patients’ experiences (phenomena). [3, 5, 13] He firstly used the method of “static understanding”, i.e., to vividly revive a patient’s experiences within the observer’s mind while thoroughly precluding any presuppositions. The next was genetic understanding, to keenly seeking their psychic connections with other experiences or events the patients presented, by empathizing, putting oneself in the patient’s situation. He also pointed out omnipresence of limits in his method of understanding pathological experiences, and introduced the distinction between understandability (comprehensibility) (Verständlichkeit) and un-understandability of the experience from the viewpoint of the observer.

**Line 75–77 in Introduction section:**

He (Jaspers) further contended that un-understandability of the experiences was characteristic of those of patients with schizophrenia.

**Line 108–113 in Introduction section:**

He (Jaspers) maintained that PDs were typical of un-understandable phenomena immediately experienced by the patients as absolutely self-evident like an intuition or revelation accompanied with radical transformation or emergence of thoughts; they were also designated as delusion proper (echte Wahnidee), from which secondary delusions evolve via secondary thought process.[14, 29]

**Comment B**

This idea of incomprehensibility (un-understandability), which is, moreover, widely criticized by the authors of post-Jaspersian psychopathology, is above all described in an intersubjective mode. In this sense, the idea of self-evaluation by patients, interesting in itself, does not seem to me to be here the most relevant way of evaluating the presence or absence of PD,

**Response to Reviewer 1’s comment B**

Because of the method used in this study, the first-person based description, this study could hardly address the issue of (un-)understandability or intersubjectivity. We described this drawback as a limitation in the previous manuscript. We have added a few words to explain the situation of this study.

**Line 585–593 in Limitations subsection:**

The fourth limitation is that the method of description applied in this study heavily depended on the self-reflections of patients. As Jaspers argued that one prominent feature of PDs was un-understandability, which is hardly recognizable without the viewpoint of interpersonal relations. The criticism of Sass and Parnas [45] stating that cognitive models in pursuing understanding from a general psychological viewpoint disregard the un-understandable area of psychotic experiences, is also relevant to this study. Future studies of primordial conditions need to remedy this limitation by incorporating other approaches such as those to focus on the relatedness to the society or others.

Concerning the question of adequacy of PD assessment of this study (“not the most relevant way of evaluation”), we admit that the PD addressed in this study were not ascertained to be exactly the same as that of Jaspers on account of its methodological restriction. Therefore, we added the limitation as follows.

**Line 581–585 in Limitations subsection:**

Third, caution is needed as to the adequacy of PD assessment in this study because it did not thoroughly examine PD accompanied characteristics such as un-understandability and abnormality in the form of experience. To make the assessment more comprehensive, it can be contributory to include these characteristics in addition to those of subjective experiences.

**Comment C**

The absence of such PD in patients with non-schizophrenic psychotic disorders (persistent disorders or paranoia, affective disorders), whereas patients with schizophrenia may present both PD and delusion-like ideas (secondary delusions). These different points are briefly mentioned in the introduction, but too quickly, whereas the interpretation and use of the notion of PD appears problematic in my opinion.

**Response to Reviewer 1’s comment C**

Although it is inferred from Jaspers’ contentions that PDs were typically seen among those with schizophrenia, which had been included as shown Response to Reviewer 1’s comment A, it is hard to confirm that they emerge specifically among the patients. Moreover, it is supposed that delusion-like ideas (secondary delusions) have less specificity as to the types of psychoses. Considering this diagnostic uncertainty, we do not suppose that it be necessary to mention this issue further in the manuscript.

**Comment D**

- what consideration and what links with PD of the bizarre characteristic of delusion, present in the criteria for schizophrenia in DSM IV (used in the present study), and its absence in DSM 5. A few words on this point could be relevant to the question of Jaspersian incomprehensibility

**Response to Reviewer 1’s comment D**

We could not find any description of the direct relatedness between PDs and bizarre delusions in “General Psychopathology” by Jaspers whereas the both seemed indirectly connected via common characteristics such as un-understandability and abnormality in the forms of experience. Instead of referring to the relationship between PDs and bizarre delusions, we have presented the connections in the revised manuscript as shown in Responses to Reviewer 1’s comments A and B.

In addition, we have briefly described the position of bizarre delusions in DSM-IV and DSM-5, and core symptoms of passivity, influence and control described in ICD-11 as follows.

**Line 79–89 in Introduction section:**

The hallmark was that he proposed the first rank symptoms (FRS), a list of particularly un-understandable psychotic symptoms, as pathognomonic signs of schizophrenia.[1] Bizarre delusions defined in the criteria of the Diagnostic and Statistical Manual of Mental Disorders, 4th Edition (DSM-IV) [16] (though absent in those of DSM-5) and the core symptoms of influence, passivity, or control experiences in the description of the 11th revision of the International Classification of Diseases (ICD-11) were included in this list.[17]

**Comment E**

The distinction between internal / external delusions, defended in the introduction, has also been widely contested in psychopathology (see Parnas and Sass, 2001, rightly cited here but unfortunately insufficiently integrated)

**Response to Reviewer 1’s comment E**

The position of this study is that of descriptive psychopathology that deals with superficial or on-surface characteristics of patients’ subjective experiences. Consequently, the internal / external distinction is based solely on perceptual characteristics of subjective experience (or psychologically assumed locations where a psychic event occurs), not on epistemological discourses or existential (or ontological thoughts addressing the relationship of the patient to him (her) self, to the world and to others in the experiences.

The drawbacks derived from not treating these issues as a whole contained in Jaspers’ PD concept, are taken up as a limitation in Limitations subsection as indicated in Responses to reviewer 1’s general comment and comment B.

**Comment F**

The mutualization of patients suffering from schizophrenia and schizoaffective disorder in the sample (I will propose to evaluate only patients with schizophrenia, as long as the presence or not of phenomena labeled as PD is not more robustly evaluated in affective disorders with psychotic characteristics,

**Response to Reviewer 1’s comment F**

We admit that PDs are generally more typically observed among patients with schizophrenia than those with other psychotic disorders. However, it cannot be denied that they are also recognized in florid psychotic conditions of patients with other psychotic disorders. Particularly, schizoaffective disorder brings about psychotic conditions of the severity and chronicity comparable to those of schizophrenia. Additionally, diagnostic criteria A concerning psychotic symptoms of schizoaffective disorder in DSM-IV and DSM-5 are the same as those of schizophrenia, indicating that there is no distinctive difference in psychotic symptomatology between the two psychotic disorders. For these reasons, we suppose that the inclusion of patients with both of the disorders in the study sample is justified.

**Comment G**

As recalled by the authors as limits, the absence of a longitudinal approach, the lack of consideration of hallucinatory phenomena.

**Response to Reviewer 1’s comment G**

We agree that longitudinal investigations are surely needed in future, which will elicit far more persuasive results concerning clinical importance of PDs than cross-sectional studies.

In addition, it is very important for future studies to include hallucinations as “primary” experiences in considering progression processes of delusions.

These drawbacks have been described in the limitation subsection.

In addition, the reason why this study did not deal with hallucinations has been made clear as follows.

**Line 222–224 in Method section:**

Hallucinations were not dealt with as origins of delusions here since Jaspers did not counted them as primary experiences of delusion, while admitting that they were possible origins of delusion-like ideas (secondary delusions) (wahnhafte Ideen).[9, 14]

**Line 579–581 in Limitations subsection:**

Second, hallucinations and bizarre delusions [37] were not dealt with in this study though they were possibly primary symptoms of delusional experiences along with PDs. [9, 14]

**Comment H**

… either a less strictly Jaspersian orientation of the central hypothesis (except for the description of the section B criteria section B of the DOAI scale), to reorient more modestly the whole on the links between the different characteristics of schizophrenic delusions, and / or a more detailed introduction on the concepts of primary delusion, to allow authors to defend their hypothesis more rigorously.

**Response to Reviewer 1’s comment H**

We appreciate the constructive proposal. On that basis, we have clarified that the methodological position of this study is restricted to descriptive psychopathology from the first person perspective of patients, and that the methodology can hardly handle the issue of un-understandability of PDs as indicated in Response to Reviewer 1’s general comment and comments A, B, E and F.

**Reviewer 2:**

**Comment A**

It is regrettable that the PANSS was chosen as a basis for comparison because its clinical relevance remains purely symptomatic and descriptive, and has been demonstrated above all in the field of pharmacology, uncritically equating the pathology of schizophrenia with what Jaspers called a cerebral mythology. In the same line, it is regrettable that the ipseity evaluation scale (EASE) proposed by Parnas, or that of Fuchs examining the world experience (EAWE) closer to a constitutive phenomenology, was not discussed by the authors to complete the fine descriptive and dynamic analysis of the first delusional symptoms.

**Response to Reviewer 2’s comment A**

We have chosen PANSS since it is one of the standard assessment scales for psychotic symptomatology, and our study group examined its reliability (Igarashi, et al, Psychiatr Clin Neurosci, 1998). It is also of note that this assessment scale derived from the different research tradition possibly had some value for testing convergent validity of DOAI. At the same time, we agree that EASE and EAWE would also have been appropriate assessments for this study, and with our reverence for these works, have added EASE and EAWE in the reference of this manuscript.

We have added the following sentence for explaining PANSS.

**Line 262–244 in Methods section:**

To assess psychotic symptoms, the Positive and Negative Syndrome Scale (PANSS) was employed, [36] which has been widely used for the assessment of patients with psychoses in clinical and research settings.

We have referred to EASE and EAWE as shown below.

**Line 85–89 in Introduction section**

The symptomology postulated by Schneider was incorporated in psychiatric evaluation systems such as Present State Examination (PSE) [18], Das AMDP-System (AMDP) [19], and recent ones of Examination of Anomalous Self-Experience (EASE) [20] and Examination of Anomalous World Experience (EAWE). [21]

**Comment B**

…The article therefore deserves to be published, with minor modifications seeking to further clarify the essential link between the initial delirious atmosphere and its subsequent encystation in the form of persistent delusion.

**Response to Reviewer 2’s comment B**

The scope of this study is within descriptions of subjective experience, and cannot reach the area of constitutive psychopathology. Therefore, the essence (nature) of the links found in this study can be discussed only insufficiently, while they are supposed to be concerning secondary processes such as judgement and thinking, which arise subsequently from PDs.

Our situation is explained as follows.

**Line 491–494 in Discussions section**

This study further identified individual relationships between PDs and characteristics of developed delusions as presented in the next subsection, which are assumed to be parts of delusion progression secondarily arising out of PDs.

**Line 540–541 in Discussion section**

The important point here is that these secondary processes appear to proceed on the basis of particular abnormalities inherent in each of PD.

**Comment C**

We suggest the authors might add a few more lines explicating a little more the actuality of the work of Jaspers to emphasize more and more its rich phenomenological posterity as a clinical epistemology bridging subjective experience, cognitive, psychopathological, and ecological conceptions of brain (see Fuchs).

Possibly to be quoted :

Fuchs, T. (2017). Ecology of the brain: The phenomenology and biology of the embodied mind. Oxford University Press.

Sass, L., Pienkos, E., & Fuchs, T. (2017). Other worlds: Introduction to the special issue on the EAWE: Examination of Anomalous World Experience. Psychopathology, 50(1), 5-9. »

**Response to Reviewer 2’s comment C**

We have much extended the description of Jaspers’ works concerning PD concept as indicated in Responses to Reviewer 1’s general comment and comment A.

As recognition of the admirable insight into the essence of patients’ subjective experience of Karl Jaspers, we have inserted the following sentence.

**Line 600–602 in Conclusion subsection**

It is also suggested from this study that further reappraisal of Jaspers’ psychopathological theses will provide some novel insights valuable for present-day psychiatric practice and research.

Fuchs’ volume is highly relevant to this study, but too far-reaching and ideal to be quoted in the manuscript, which is incomparable to our study that is only wandering around the most basic area of descriptive psychopathology. Thus, we would like to refrain from citing this work.

The changes are indicated by red letters in the file of “Revised Manuscript with track changes.”

Lastly, we would like to express our profound appreciation for the reviewers’ considerate and constructive suggestions and proposals for improving our manuscript.
