# Supplementary material for: Delusion progression process from the perspective of patients with psychoses: A descriptive study based on the primary delusion concept of Karl Jaspers
Source: PLoS One. 2021 Apr 27;16(4):e0250766. doi: 10.1371/journal.pone.0250766 (PMC8078756; doi:10.1371/journal.pone.0250766)
Supplement: Attachment — Submitted filename: Responses to the decision R2.docx [file pone.0250766.s005.docx]

Responses to comments of the editor, and changes made on that basis

(Principally, the changes are indicated by red letters in this report.)

PONE-D-20-21497R2

Delusion progression process from the perspective of patients with psychoses: A descriptive study based on the primary delusion concept of Karl Jaspers

Comment A

The standard of English is not fully acceptable. If possible, please arrange for the paper to be read and corrected by a native English speaker.

Response to Comment A

The revised manuscript was checked by an English editing service company (the check certificate is therewith up-loaded.)

Comment B

It would make sense to use the term understandable/ ununderstandability instead of incomprehensible/ incomprehensibility, as this is the term customarily used to refer to Jaspers' thinking on this point.

Response to Comment B

Which word pair is used in the manuscript is a delicate matter, as the editor noted. We selected “(un)understandability” and “understanding” for the revised manuscript instead of “(in) comprehensibility” and “understanding”, which were used in the translation of “General psychopathology (1959 edition)” by Hoenig J and Hamilton MW. We believe “understandability” and “understanding” are more understandable as they parallel the derivation of “Verstantlichkeit” from “Verstehen.”

We examined several books, and found that both “understandability” and “comprehensibility” are comparably commonly used. The former is used in "Symptoms in the Mind: An Introduction to Descriptive Psychopathology, 3rd Ed. (2003)" by Sims A. In “One Century of Karl Jaspers' General Psychopathology (2013)” edited by Stanghellini & Fuchs, and “Karl Jaspers’ Philosophy and Psychopathology (2014)” edited by Fuchs Breyer and Mundt, and both word pairs were used by different authors.

Comment C

The editor requested to correct the following sentence to more precisely describe our present understanding of delusion.

“However, delusions the patients present with are endo-products of concurrent disturbances of mental activities such as thinking, perception, memory, and aﬀect.[2, 4, 6, 7, 9, 10]”

Response to Comment C

We revised this sentence by merging it with the next sentence as follows.

” However, capturing the background of changes in delusions is considered infeasible [9-11] because they are thought to be influenced by diverse disturbances of mental activities such as thinking, perception, memory, and affect [2, 4, 6, 7, 12, 13].”

Comment D

The editor noted the misspelling of “endo-products”

Response to Comment D

The part containing this word was deleted as shown above.

Comment E

The editor noted that bizarre delusions were not included as first rank symptoms by Schneider.

Response to Comment E

We made a mistake in believing that the bizarre delusions were partly successors of Schneider’s RFSs. We added a reference and replaced the sentences with the following for a more precise description:

“Some FRSs may be considered samples of bizarre delusions defined by the criteria for schizophrenia in the Diagnostic and Statistical Manual of Mental Disorders, 4^th^ Edition (DSM-IV) [16 (Cermolacce M, Sass L, Parnas J. What is bizarre in bizarre delusions? A critical review. Schizophr Bull. 2010;36(4):667-79.)].”

Comment F

The editor requested a stronger literature background as to whether delusional memory was included in primary delusions in addition to the 1913 edition of Jaspersʼ General Psychopathology.

Response to Comment F

Jaspers did not use the term “delusional memory” in the 1913 and 1959 editions of the book, but wrote “...which (delusion content) is then confirmed by a vivid memory...(P. 103, Vol. I of the 1959 edition)”.

However, the definition of Jaspers’ PDs written by Sims A [6], which included delusional memory, was included in the first paragraph in P. 123, "Symptoms in the Mind: An Introduction to Descriptive Psychopathology, 3rd Ed. (2003)": the 4 types of PDs are “(a) autochthonous delusion (delusional intuition), (b) delusional percept, (c) delusional atmosphere, and (d) delusional memory.”

Therefore, we revised the respective part on Pages 7-8 of the manuscript as shown below.

“The PDs referred to by Jaspers were delusional awareness or mood (atmosphere), delusional perception, and delusional idea (delusional intuition (sudden notion) and delusion confirming memory (delusional memory)) [6 (the book of Sims), 12 (Jaspers, 1913)].”

Comment G

The editor noted that the part referring to the cognitive theory or cognitive model of delusions proposed by Beck A et al., Garety PA et al., and Moritz S et al. was essentially outside the clinical descriptive/phenomenological approach to delusions, which is the focus of the paper, and made two proposals: The first was to omit this part, and the second was to add a paragraph in the discussion on the degree to which the phenomenological approach and our findings concur with these cognitive/ cognitive therapy-based approaches.

Response to Comment G

Although the models of cognitive theories are from different veins of research, the characteristics of subjective experience of patients with delusions is the important basis of both research areas. For example, the study series by Garety PA et al. utilized the assessment scale for phenomenology of delusions [22 (Garety PA, Hemsley DR. Characteristics of delusional experience. Eur Arch Psychiatry Neurol Sci. 1987;236(5):294-8.)]. Referring to these theses in our descriptive study should have great value.

We deleted a reference to the work of Beck et al. because it required complicated discussions to connect it to our study. On the other hand, we added the following paragraph connecting the findings of this study to those from Garety et al. and Monitz et al. regarding PDs and delusion progression on Page 37.

“These findings are also consistent with those of the cognitive models. The distinction of cognitive features between Persecutory delusions [39, 40 (Freeman D et al.)] and Grandiose delusions [41 (Garety PA et al.)] is parallel to the delusion theme types found in this study, and the 2-stage cognitive model [31 (Moritz S et al.)] corresponds to the progression from PDs to secondarily developed delusions. PDs may represent cognitive alterations postulated by cognitive models to be factors for delusion formation.”

Comment H

The editor mentioned that Kapur’s salience hypethesis had little connection with the descriptive nature of this study, and requested to use it in the proper context.

Response to Comment H

We would like to stress that it was Kapur S himself who stressed the importance of phenomenological features of delusional mood and appraised them as typical of salience phenomena, which has subsequently become a large topic in descriptive psychopathology, and encouraged researchers to write several papers, such as Mishra AL et al. [42] and Sass L et al. [43], stressing the value of descriptive psychopathology.

We amended the sentence on Page 9 to stress the important role of the phenomenology in the hypothesis.

“By focusing on phenomenological features of delusional mood, Kapur [32] proposed the aberrant salience hypothesis that chaotic disinhibition of subcortical and prefrontal dopamine release in acute schizophrenic psychosis is associated with excessive salience attributed to otherwise irrelevant stimuli.”

Comment I

The editor questioned the justifiability of PANSS cluster scales used in the previous manuscript, and recommended using more intuitive and reader-familiar models, such as that of Wallwork RS, et al., which was constructed by referring 29 studies.

Response to Comment I

The model used in the previous manuscript was constructed in the study of White et al. (PANSS study group: Empirical assessment of the factorial structure of clinical symptoms in schizophrenia. A multisite, multimodel evaluation of the factorial structure of the Positive and Negative Syndrome Scale. Psychopathology. 1997;30(5):263-74.), which was conducted by researchers, including some founders of the PANSS, applied a patient sample with the maximal size (N=1233), and tested 20 models derived from the PANSS factor analytic studies available at the time. Although the study of White et al. has strong points, it is old. Therefore, we applied the model of Wallwork RS et al. recommended by the editor.

We revised the results of correlation analyses concerning PANSS assessment and their related descriptions in the Methods and Discussion sections.

In addition, we clarified that the PANSS factor scores and relevant individual item scores were used for examining the convergent validity and characterizing DOAI scales in the following sentence on Page 18:

“To examine the convergent validity and clinical significance of DOAI scales of PDs and delusional themes and features, correlation analyses of these scores and those of the consensus PANSS factors proposed by Wallwork et al. [38] and relevant PANSS individual items were performed by calculating Pearson’s product-moment or Spearman’s rank-order correlation coefficients.”

Comment J

The editor requested additional explanation about the high percentage of subjects experiencing PDs

“One hundred subjects (93%) reported that they had experienced at least one PD explained as an origin of delusions with a “Mild” or higher than “Mild” level.”

Response to Comment J

This high frequency may have been caused by the study setting of semi-structured interview, in which PDs were inquired about in connection with the specific delusion. If PDs are not examined in this manner, the frequency will be smaller. Therefore, we stressed this particular assessment situation in the first paragraph of the Discussion section as follows:

“Based on the subjects’ self-reflections about the origins of delusional experiences inquired about in a semi-structured interview setting, the present study demonstrated that the majority of subjects recognized PDs as origins of delusions they held concurrently. This supports the PD-based hypothesis of delusion development: PDs are delusion-preceding phenomena, interpretable as those of the early stages of delusion development [3, 10, 29]. A unique point of this study was that it approached the primary experience of delusions by investigating the experiences that subjects answered as their origins”

Comment K

The editor requested clarification of what period of delusional experiences was investigated.

Response to Comment K

The delusional experiences dealt with in this study were those the subjects were concurrently holding or experiencing.

To stress this matter, we added a sentence and phrase that indicate the period of experiencing delusions on P. 12 and P. 34, respectively.

“In Section A, the symptomatic severity of delusions during the past 3 months for individual themes was assessed.”

“the present study demonstrated that the majority of subject recognized PDs as origins of delusions that they held concurrently.”

Comment L

Delusional memory was present at some point in nearly half of the sample. This seems very frequent for a symptom that is usually considered to be rare. Please comment on this finding in the discussion.

Response to Comment L

As presented in the response to Comment J, the high percentage of subjects experiencing delusional memory and other PDs was due to the investigation setting, in which they were inquired about them in connection with specified delusions.

Comment M

The editor requested to improve the clarity of the discussion by saying “Please review for clarity. In particular, when you make assertions based on findings of the study, it would be helpful to make clear what the relevant findings were.”

Response to Comment M

For further clarification, we added several phrases referring to the findings of this study. We suppose that this comment was mainly regarding the descriptions of PDs and delusion progression subsection, where a number of results from the MANCOVA regressions and correlation analyses were referred to. The section of PDs and delusion progression with our amendments, although lengthy, is shown below.

“**PDs and delusion progression**

MANCOVA regression analyses in this study revealed many associations of PDs with delusion themes and features. The analysis of delusion themes found 2 groups of their associations with PDs, which can be assumed to be types of progression: one from D-Percep and D-Mood to Persecutory delusions, and the other from D-Intui and/or D-Memo to Grandiose, Hypochondriac, and Being assisted or loved delusions. These 2 types of associations are easily recognizable in case vignettes presented in previous studies of descriptive psychopathology. For example, 4 of 6 case vignettes of delusional perception that Jaspers presented [12] contained features of Persecutory delusion or ideas, whereas other delusion themes were absent, and both of the case vignettes of D-Intui and D-Memo [12] included features of grandiosity.

These types of progression are comprehensible from 2 points of view: their location (outside vs inside) and thought process characteristics. Persecutory delusions often have outside origins, reflected by their associations with D-Percep and D-Mood. The thought processes involved are those mainly related to the abnormal interpretation of external stimuli, hence the name delusional interpretation [2, 6] or paranoid interpretation [39]. In addition, this study revealed that Persecutory delusions are considerably correlated with negative affect, such as anxiety and depression, as demonstrated in previous studies [13, 40]. Therefore, this progression was considered externally initiating and a therewith invading process.

In contrast, the second type of progression was considered to be initiated and fortified as a within-mind process. Grandiose, Hypochondriac, and Being assisted or loved delusions were associated with D-Intui and/ or D-Memo, internally evolving types of PDs, but not with D-Percp and D-Mood. In particular, Being assisted or loved and Hypochondriac delusions had negative associations with D-Percep or D-Mood, which suggests that these delusions are furthered by discarding stimuli from the external environment. They were also correlated with strong confidence (lack of doubt) in their delusions. This type of progression may be related to disturbed thought processes, as suggested by the correlation of D-Intuit with PANSS Disorganized/ concrete factor. This factor is related to disturbance in concept integration and concentration [38], which is different from that of paranoid interpretation referred to above. In addition, D-Memo, which was associated with Grandiose delusions, is a phenomenon that is maintained internally by recall repetition even though its initiated experience may have had external origins [3]. Thus, progression of the second type is considered to be an internally precipitating and thereon consolidating process.

Similarly, the connections of PDs with delusion features are of clinical relevance. S/G, a feature of highly-developed delusions, was furthered by D-Percep and D-Intui based on MANCOVA regression, suggesting that both external percept-initiated and internally-precipitated processes have additive effects on S/G. Almost the same point, i.e., D-Percep, D-Intui, and thought disorder are important in delusion system generation, was stressed by Schneider [1].

The association of D-Mood with NAR may be explained by previous descriptions that D-Mood is characterized by the domination of intense negative affect [12, 14]. NAR can also be interpreted to be derived from D-Mood.”
