# Supplementary material for: Delusion progression process from the perspective of patients with psychoses: A descriptive study based on the primary delusion concept of Karl Jaspers
Source: PLoS One. 2021 Apr 27;16(4):e0250766. doi: 10.1371/journal.pone.0250766 (PMC8078756; doi:10.1371/journal.pone.0250766)
Supplement: Attachment — Submitted filename: Responses to the editors comments R3.docx [file pone.0250766.s006.docx]

Responses to comments of Dr. Peter John McKenna, Academic editor

PONE-D-20-21497R2

Delusion progression process from the perspective of patients with psychoses: A descriptive study based on the primary delusion concept of Karl Jaspers

The editor kindly advised in the judgement letter on 17 March, 2021 that we need to amend three points in the manuscript.

Additionally, through personal e-mail communications, the editor provided a myriad of corrections to dramatically improve the quality of English writing, and indicated eight points we should reconsider or amend to arrange the logical framework in the manuscript.

The comments and our response to them are presented below. Principally, the changes are indicated by red letters in this report.

1. The editor wondered if the following sentence in Line 87, Page. 6 lacks clarity. We have attempted to make it more informative.

The previous sentence was ʻIn addition, Kendler et al. [21] and Oulis et al. [26] performed factor analytic studies on delusion characteristics, and extracted factors, such as delusional involvement and cognitive disintegration; however, their results were not consistent.ʼ

We have amended this one as follows in Line 97~101, Page 7

ʻIn addition, Kendler et al. [21] and Oulis et al. [26] performed factor analytic studies on delusion characteristics, and extracted some principal features such as those of influence by delusion and disturbed thought process; however, the results of these two studies did not converge well. ʼ

2. The editor requested a more exact explanation for the selection of PD items that were used in our study, and kindly exemplified a sentence for correction.

The previous sentence was ʻThe PDs referred to by Jaspers were delusional awareness or mood (atmosphere), delusional perception, and delusional idea (delusional intuition (sudden notion) and delusion confirming memory (delusional memory)) [6, 12].ʼ

We have made the following corrections on the basis of the editor’s quite pertinent proposal about the definition of PD types used in the study in Line 110~114, Page 7.

ʻThe PDs referred to by Jaspers [12] were delusional awareness or mood (atmosphere), delusional perception, and delusional idea (delusional intuition or sudden notion) and recollection of delusion confirming memory (Sims [6] later paraphrased as delusional memory, a term Jaspers did not explicitly use).

The additional corrections are made in Line 189~193. Page 12 in Assessment of PDs and delusion characteristics subsection.

‘This section enquired about the 4 PD types that Sims [6] defined based on description by Jaspers [12]: B-1. Delusional perception (D-Percep) (Wahnwahrnehmung), B-2. Delusional memory (memory recall) (D-Memo) (Wahnerinnerung), B-3. Delusional mood (D-Mood) (Wahnstimmung), and B-4. Delusional intuition (D-Intuit) (Wahneinfall).’

3. The editor pointed out that the subject selection criterion of “(iii) not having a prominent organic cognitive disorder or intellectual disability that makes the assessment difficult” was ambiguous.

We have replaced this criterion with the following one in Line 164~165. Page 11 to improve its clarity.

‘(iii) not having a comorbid diagnosis of dementia and other cognitive disorders, or mental retardation’

The followings are the editor’s additional proposals made through e-mail communications and our responses to them.

4. The editor requested to explain more about the sentence in Page 4 ‘The associations of PDs with delusion characteristics can be interpreted in terms of intensification and generalization of pathological cognitive alternations.’

We have changed this sentence to the following one in Line 46~49, Page 4 in Abstract.

“The associations between PDs and delusion characteristics can be interpreted in terms of progression processes of delusions, which are seen as intensification and generalization of cognitive and affective pathologies in PDs.”

We have also replaced the two sentences that were after paragraphs presenting the findings of PD-delusion characteristic associations with the aim to summarize the findings, with the following two sentences in Line 556~559, Page 37.

“These findings of the associations presented above can be interpreted in terms of intensification and generalization of cognitive and affective characteristics recognized in PDs. The important point here is that the formative process of secondary delusions proceeds while maintaining continua with abnormalities inherent to each PD.”

5. The editor questioned if the study of Markova & Berrios [30] specifically addressed delusion symptoms concerning the sentence in Line 126~128, Page 8.

We have corrected the sentence to indicate that the symptoms were psychotic ones in general, not specific to delusions, as indicated in the following sentence.

“Markova & Berrios [30] outlined a formative process of psychotic symptoms from experiencing something unnamed to expressing them as symptoms in verbal communication.”

6. The editor requested to indicate clearly that ‘S1 TEXT’ is a supplementary information.

We have added a sentence of “This schedule (DOAI) is included in the supporting information of this study as S1 TEXT.” in Line 176~177, Page 12.

7. The editor pointed out that the expression ‘clearly or concurrently present’ in Table 2 is unclear.

We have deleted words of “or concurrently.” The words were solely for frequency items for B-1 to B-4. We suppose that the additional explanation it in the note fot Table 2 would suffice to indicate this situation.

8. The editor advised that a reference of Table 6 be put somewhere around the table.

We have added a reference of this table in Line 488, Page 32.

9. The editor requested to make the sentence ‘Overall, this second type of progression may be considered to reflect an internally precipitating and consolidating process.’ more easily understandable.

We have replaced it with the one “Overall, this second type of progression can be considered to be an internally evolved and thereon consolidated process.” in Line 542~543, Page 35.

10. The editor advised that we add some explanation to, or delete the sentence in Line 553~555, Page 36 with the comment that memory is not particularly unsusceptible to all kinds of internal and external influences.

We have changed this sentence as follows.

“One explanation for this finding (D-memo is only a significant factor for patients’ delusional judgements) could be that delusional memory is often held more firmly and endurably than other PDs, which may result in its having a stronger influence on the judgements.”

11. The editor commented that “alterations of cognition and emotion” in Page 38 is unclear in this context, and wondered whether it was indispensable.

We suppose that this part is indispensable since it represents the findings from the latter part of MANOVA analyses. Therefore, we have attempted to indicate clearly the context where it is situated in the sentences in Line 574~577, Page 38 as follows.

“Secondly, this study suggests that there is a wide variation in the progression processes and composing factors of delusions of persecutory and grandiose delusions, pathological affective responses and cognitive disturbance such as delusional systematization and lack of insight into delusion.”

Additionally, all “inquiries” have been replaced with “enquiries” in the manuscript.
